# Genome-wide identification and expression patterns analysis of the *RPD3/HDA1* gene family in cotton

**DOI:** 10.1186/s12864-020-07069-w

**Published:** 2020-09-18

**Authors:** Jingjing Zhang, Aimin Wu, Hengling Wei, Pengbo Hao, Qi Zhang, Miaomiao Tian, Xu Yang, Shuaishuai Cheng, Xiaokang Fu, Liang Ma, Hantao Wang, Shuxun Yu

**Affiliations:** State Key Laboratory of Cotton Biology, Institute of Cotton Research of Chinese Academy of Agricultural Sciences, Anyang, 455000 Henan China

**Keywords:** *Gossypium*, Histone deacetylases, Expression patterns, Abiotic stress, Early maturity

## Abstract

**Background:**

Histone deacetylases (HDACs) catalyze histone deacetylation and suppress gene transcription during various cellular processes. Within the superfamily of HDACs, *RPD3/HDA1*-type HDACs are the most studied, and it is reported that *RPD3* genes play crucial roles in plant growth and physiological processes. However, there is a lack of systematic research on the *RPD3/HDA1* gene family in cotton.

**Results:**

In this study, genome-wide analysis identified 9, 9, 18, and 18 *RPD3* genes in *Gossypium raimondii, G. arboreum*, *G. hirsutum*, and *G. barbadense*, respectively. This gene family was divided into 4 subfamilies through phylogenetic analysis. The exon-intron structure and conserved motif analysis revealed high conservation in each branch of the cotton *RPD3* genes. Collinearity analysis indicated that segmental duplication was the primary driving force during the expansion of the *RPD3* gene family in cotton. There was at least one presumed cis-element related to plant hormones in the promoter regions of all *GhRPD3* genes, especially MeJA- and ABA-responsive elements, which have more members than other hormone-relevant elements. The expression patterns showed that most *GhRPD3* genes had relatively high expression levels in floral organs and performed higher expression in early-maturity cotton compared with late-maturity cotton during flower bud differentiation. In addition, the expression of *GhRPD3* genes could be significantly induced by one or more abiotic stresses as well as exogenous application of MeJA or ABA.

**Conclusions:**

Our findings reveal that *GhRPD3* genes may be involved in flower bud differentiation and resistance to abiotic stresses, which provides a basis for further functional verification of *GhRPD3* genes in cotton development and a foundation for breeding better early-maturity cotton cultivars in the future.

## Background

DNA combines with nuclear proteins to constitute the chromatin, which is responsible for storing genetic and directive information in eukaryotic cells. Chromatin is highly arranged and mainly composed of nucleosomes, which are formed by approximately 147 bp of DNA and an octamer organized by the four core histone proteins_H3, H4, H2A, and H2B [1]. Gene expression in eukaryotes involves a complicated interaction, which is controlled not only by the DNA sequence but also by epigenetic events. Epigenetic mechanisms mainly consist of histone modification and DNA methylation, and play an important role in the regulation of gene expression. In general, histone posttranslational modifications, including methylation, acetylation, phosphorylation, ADP-ribosylation and ubiquitination, occur at the N-terminal of histones [2], and these changes facilitate the binding of other proteins to DNA, resulting in synergistic or antagonistic regulation of gene transcription [3, 4] . Among the several histone modifications, histone acetylation is a reversible process that plays essential roles in epigenetic regulation. The acetylation of core histones is catalyzed by histone acetyltransferases (HATs) to promote transcriptional activation, whereas deacetylation is regulated by histone deacetylases (HDACs) that drive the transcriptional suppression [5]. HDACs deacetylate the lysine residues of N-terminal histone tails, resulting in the repression of gene expression [6].

HDACs are involved in a large amount of biological processes associated with plant growth and development [7–9]. Based on sequence homology to yeast HDACs, HDACs in plants are divided into three main categories: reduced potassium dependency 3 / histone deacetylase 1 (*RPD3/HDA1*), histone deacetylase 2 (*HD2*), and silent information regulator 2 (*SIR2*) [7, 10, 11]. *RPD3/HDA1*-type histone deacetylases, which are homologous to yeast *RPD3* and *HDA-1*, belong to a large family, and they require zinc ions to catalyze activity; the HDAC inhibitor trichostatin A (TSA) or sodium butyrate can inhibit their enzymatic activities [7]. The *Arabidopsis RPD3/HDA1* gene family is further classified into three groups. Class I includes *HDA6*, *HDA7*, *HDA9*, and *HDA19*; class II includes *HDA5*, *HDA15*, and *HDA18*; and *HDA2* is the only member of class III [7, 8]. The other genes of *PRD3/HDA1* family are unclassified in *Arabidopsis*.

Over the past 20 years, *RPD3/HDA1*-type HDACs (call *RPD3* for short below) have been studied extensively as global regulatory factors playing essential roles in a series of plant growth and development processes and the response to various environmental stresses [8, 12–14]. In *Arabidopsis*, it has been reported that *AtHDA19* was involved in various developmental processes, including flowering time, circadian clocks functions, and seed development [15, 16]. Additionally, *AtHDA19* might regulate gene expression related to jasmonic acid and ethylene signaling pathways in response to wounding and pathogen infection [17]. In maize, the expression patterns of the three *ZmPRD3* genes *ZmRpd3/101*, *ZmRpd3/102*, and *ZmRpd3/108* showed widespread expression in all investigated corn organs. Furthermore, the gene products could be detected in all cellular parts at specific stages such as kernel, shoot, and anther developmental periods [18]. In rice, *HDA705* responded to ABA and abiotic stresses, and its expression was induced by JA. In addition, the expression of *HDA702* and *HDA704* was significantly induced by SA, JA, or ABA [19, 20]. These findings indicate that the *RPD3* members play an important regulatory role in plant development and in the response to various stresses and plant hormones.

Cotton is one of the most important economic crops in China with an essential role in the national economy. Early maturity and stress resistance are vital target traits of cotton breeding. Over the past two decades, the *RPD3* genes have been intensively studied, and some progress has been made in *Arabidopsis* and some other crops. However, there is a lack of systematic research on the *RPD3* gene family in cotton. Thus, it is necessary to explore the potential functions of *RPD3* genes in cotton. In our study, the protein sequences of cotton *RPD3*-type HDACs were predicted by genome-wide identification and the phylogenetic tree, gene structure, conserved motif, protein domain, expression profiles, and preliminary functions were comprehensively analyzed. The information gained for *GhRPD3* provides a reference for further exploration of the possible functions of *RPD3* genes in cotton growth and development.

## Results

### Identification of *RPD3* genes in nine species

In this study, a total of 108 *RPD3* protein sequences from nine species were identified after eliminating redundant sequences, and they are named by the position on the chromosome. The corresponding relationship between gene ID number and gene name is shown in Additional file 1: Table S1. A total of 18 genes (*GhHDA1*-*GhHDA18*) containing Hist_deacetyl (PF00850) domains were identified from *G. hirsutum*; 9 genes were located on the At genome, and 9 genes were mapped on the Dt genome. Furthermore, 18 genes (*GbHDA1*-*GbHDA18*) from *G. barbadense*, 9 genes (*GaHDA1*-*GaHDA9*) from *G. aboreum*, and 9 genes (*GrHDA1*-*GrHDA9*) from *G. raimondii* were detected. Tetraploid cotton possessed twice as many *RPD3* genes as diploid cotton, indicating that no *RPD3* cotton gene was lost in the process of polyploidy. The numbers of *RPD3* genes in the other five species were 10 (*Arabidopsis*), 14 (*Oryza sativa L.*), 11 (*Populus trichocarpa*), 8 (*Theobroma cacao*), and 11 (*Zea mays L.*). The *GhRPD3* protein length ranged from 232 to 635 aa with an average of 459 aa. The physicochemical parameters showed that the isoelectric point (pl) of *GhRPD3* proteins varied from 4.47 to 8.65 with an average value of 5.68, and the molecular weight of *GhRPD3* proteins varied from 25.79 to 73.01 kDa with an average value of 51.21 kDa. The subcellular localization results indicated that most of the *GhRPD3* genes were located in cytoplasmic (10) or nuclear (8), suggesting that *GhRPD3* genes might possess multiple regulatory functions (Table 1). The predicted length, pI, MW and subcellular localization of the *RPD3* proteins in other eight species are shown in Additional file 1: Table S1.

**Table 1:** Physicochemical parameters of 18 *RPD3* genes in *G. hirsutum*

Name	Gnen ID	Protein Length	Protein pI	Protein MW (kD)	Subcellular localization
*GhHDA1*	*Ghir_A01G001410.1*	499	4.9676	56.18	Nuclear
*GhHDA2*	*Ghir_A03G007210.1*	471	5.076	53.09	Nuclear
*GhHDA3*	*Ghir_A03G008200.1*	655	5.325	73.01	Cytoplasmic
*GhHDA4*	*Ghir_A03G018610.1*	351	4.4737	39.55	Cytoplasmic/Nuclear
*GhHDA5*	*Ghir_A05G039610.1*	449	6.9085	48.66	Mitochondrial/Chloroplast
*GhHDA6*	*Ghir_A09G010210.1*	429	4.8969	49.08	Cytoplasmic/Nuclear
*GhHDA7*	*Ghir_A12G027820.1*	574	6.3108	63.26	Cytoplasmic
*GhHDA8*	*Ghir_A13G019980.1*	232	6.5919	25.79	Plasma Membrane
*GhHDA9*	*Ghir_A13G023460.1*	368	5.3373	40.37	Cytoplasmic/Chloroplast
*GhHDA10*	*Ghir_D01G001410.1*	499	4.9676	56.26	Nuclear
*GhHDA11*	*Ghir_D02G019970.1*	465	5.1309	52.65	Nuclear
*GhHDA12*	*Ghir_D03G010660.1*	635	4.8889	71.02	Cytoplasmic
*GhHDA13*	*Ghir_D03G011510.1*	471	5.1489	53.06	Nuclear
*GhHDA14*	*Ghir_D04G003510.1*	443	6.9591	47.95	Chloroplast/Mitochondrial
*GhHDA15*	*Ghir_D09G009940.1*	429	4.8371	49.11	Cytoplasmic/Nuclear
*GhHDA16*	*Ghir_D12G027930.1*	579	6.1788	63.80	Cytoplasmic
*GhHDA17*	*Ghir_D13G020760.1*	331	8.6517	37.28	Plasma Membrane
*GhHDA18*	*Ghir_D13G024090.1*	380	5.648	41.63	Cytoplasmic

### Phylogenetic analysis of the *RPD3* gene family

**Fig. 1:**
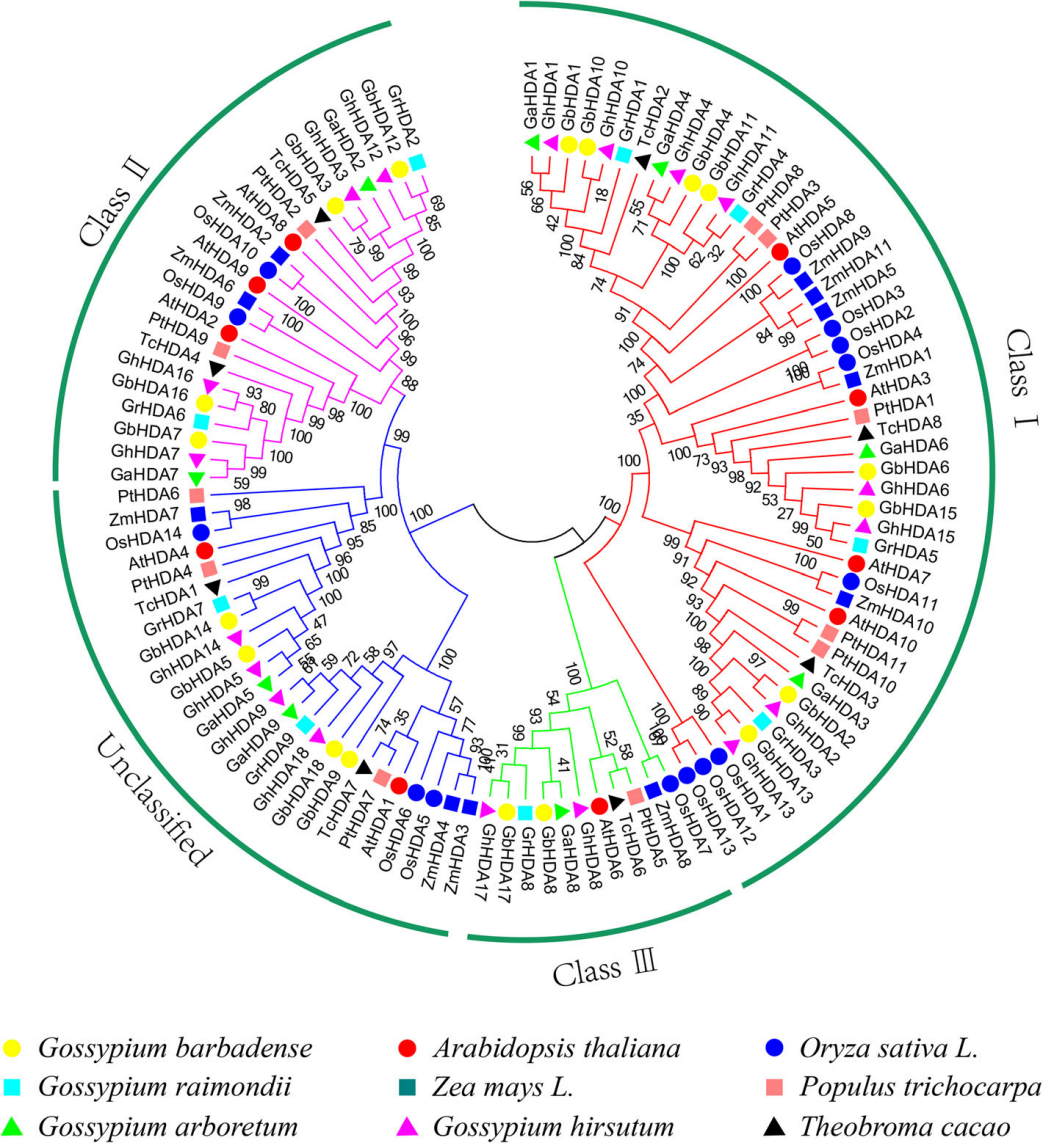
Neighbor-joining phylogenetic tree of *RPD3* gene family. The 108 predicted *RPD3* proteins from *G. hirsutum*, *G. arboreum*, *G. barbadense*, *G. raimondii*, *A. thaliana, P. trichocarpa*, *T. cacao*, *Oryza sativa*, and *Zea mays* were aligned using ClustalW, and the neighbor-joining (NJ) method was used to construct this unrooted phylogenetic tree using MEGA 7.0 program with 1000 bootstrap repetitions. Four subfamilies are represented by the different colored lines

A total of 108 identified *RPD3* protein sequences from *G. raimondii* (9), *G. arboreum* (9), *G. hirsutum* (18), *G. barbadense* (18), *A. thaliana* (10), *T. cacao* (8), *Oryza sativa* (14), *Zea mays* (11) and *P. trichocarpa* (11) were employed to construct an unrooted phylogenetic tree using the neighbor-joining method for investigating the evolutionary relationships of *RPD3* proteins. The *RPD3* proteins were phylogenetically classified into 4 subfamilies (Class I, Class II, Class III, and unclassified) according to the formulated subfamilies in *Arabidopsis* [7]. The Class I subgroup was the largest subfamily with 49 *RPD3* genes, whereas the Class III subgroup has the fewest members, only containing one gene in seven diploid species genomes and two genes in two tetraploid cotton genomes (Fig. 1). Among these four classes, each subfamily contained *RPD3* genes from all nine species, indicating this gene family was relatively conserved in different species during evolution.

### Exon-intron structure and conserved motif analysis

**Fig. 2:**
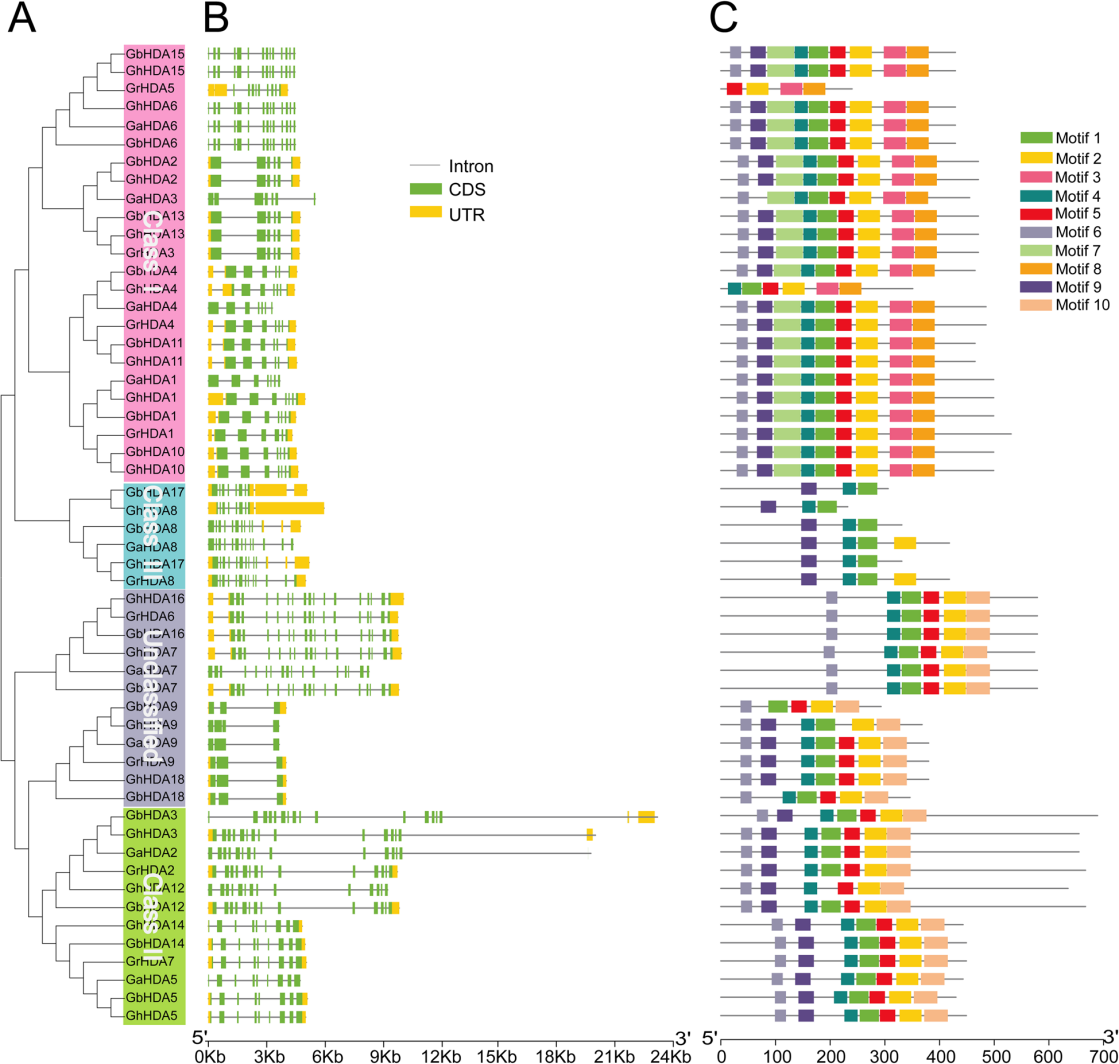
Phylogenetic relationships, exon-intron structure, and conversed motif analysis of cotton *RPD3* genes. **a** A neighbor-joining phylogenetic tree of 54 cotton *RPD3* genes was generated using the MEGA7.0 program; (**b**) Exon-intron structure analysis of 54 cotton *RPD3* genes. The UTRs, exons, and introns are represented with yellow boxes, green boxes, and black lines, respectively; (**c**) The 10 conversed protein motifs of *RPD3* genes are indicated by different colored boxes

The domains of the *RPD3* sequences in cotton were investigated and exhibited according to the results of the SMART database using TBtools, revealing that all cotton *RPD3* genes contained a Hist_deacetyl domain (Additional file 2: Table S2 and Additional file 3: Figure S1). An unrooted phylogenetic tree with the predicted cotton *RPD3* genes was constructed (Fig. 2a), and then exon-intron structure (Fig. 2b) and conserved motifs (Fig. 2c) were analyzed to better understand the similarity and differences of cotton *RPD3* members. The results showed that the length of *RPD3* cotton genes was relatively conserved in Class I and Class III, but there were twelve longer sequences in Class II and the unclassified group. The *RPD3* cotton genes included from 3 to 17 exons and most *RPD3* genes (48/54) contained more than five exons (Additional file 4: Table S3), which might be associated with the diversification of their functions. In terms of the distribution of motifs, most *RPD3* cotton genes belonging to the same subfamily showed a similar motif composition, except in the unclassified group (Fig. 2c). Most Class I subfamily members contained 9 motifs, whereas *GrHDA5* and *GhHDA4* contained 4 and 6 motifs, respectively. Class III subfamily genes had three or four motifs, and most Class II subfamily members possessed 7 motifs, except for *GhHDA12* with 6 motifs. There were differences in the exon-intron structure and motif arrangement among the four categories, but they were highly conserved on the same branches, indicating that the *RPD3* members classified into the same branch might perform a relatively conserved function in cotton growth and development.

### Chromosomal distribution, gene duplication and selection pressure

**Fig. 3:**
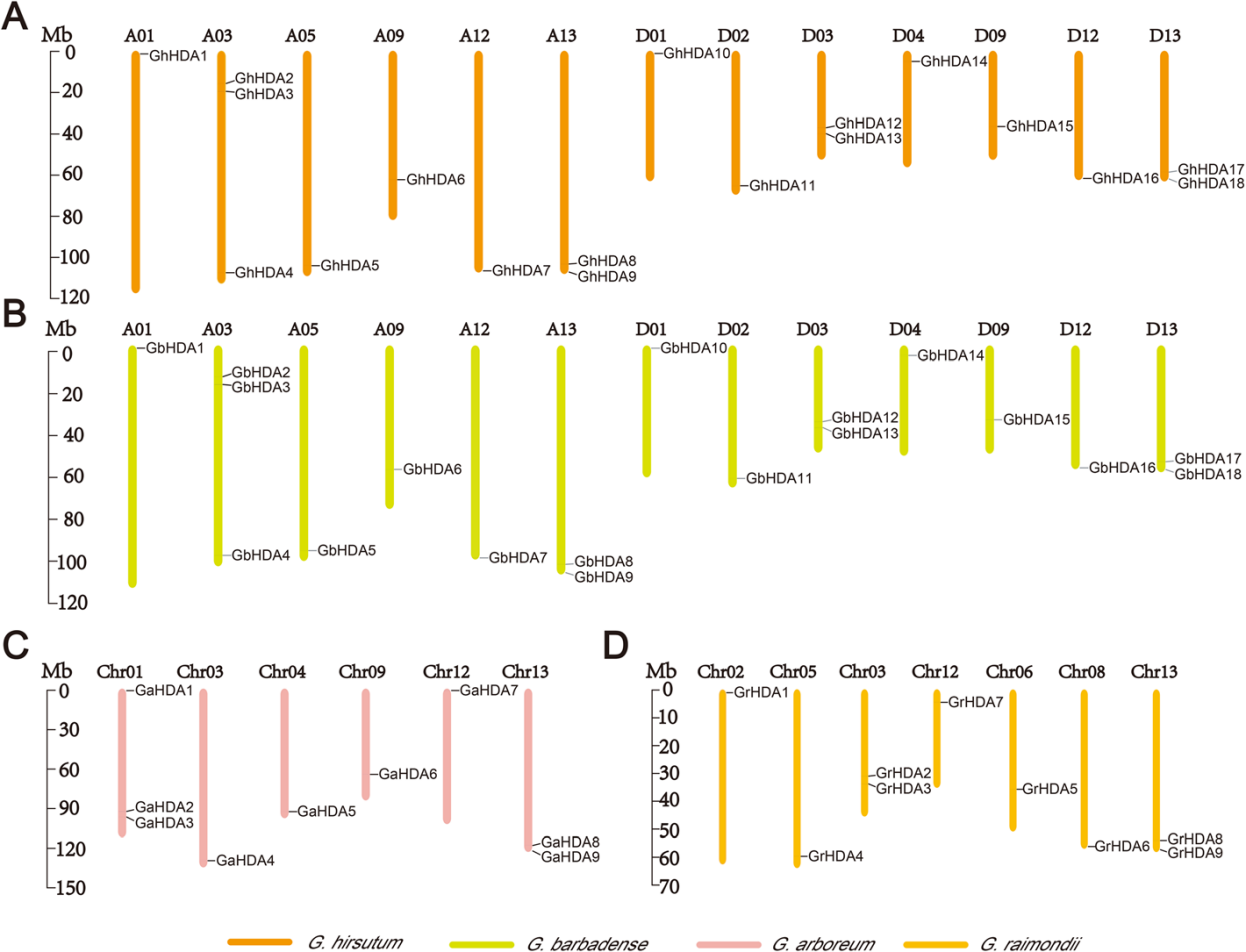
Chromosomal distribution of cotton *RPD3* genes. **a**, **b**, **c** and **d** represent the chromosomal location of *RPD3* genes from *G. hirsutum* (**a**), *G. barbadense* (**b**), *G. arboreum* (**c**), and *G. raimondii* (**d**), respectively. The chromosome number is shown on the top of each chromosome. The scale bars represent the length in mega bases (Mb)

The chromosomal distributions of *GrRPD3*, *GaRPD3*, *GbRPD3*, and *GhRPD3* genes were visualized according to the genomic position of 54 cotton *RPD3* genes (Additional file 5: Table S4 and Fig. 3). In *G. hirsutum*, 18 *GhRPD3* genes were unevenly mapped on 13 chromosomes. A03 contained the most *GhRPD3* genes (3), whereas the other 12 chromosomes only contained one or two *GhRPD3* genes (Fig. 3a). The chromosomal distribution of 18 *GbRPD3* genes in *G. barbadense* was similar to that of *GhRPD3* genes in *G. hirsutum* (Fig. 3b). In *G. arboreum*, 9 *GaRPD3* genes were unevenly located on 6 chromosomes. Chr01 and Chr13 contained three and two *GaRPD3* genes, respectively, and the other 4 chromosomes contained only one *GaRPD3* gene (Fig. 3c). In *G. raimondii*, the chromosomal distribution of 9 *GrRPD3* genes was highly consistent with the corresponding D subgenome of *G. hirsutum* (Fig. 3d), showing conserved numbers and chromosomal distribution of *RPD3* genes between diploid and tetraploid cotton species. In addition, the lopsided chromosomal distribution of the cotton *RPD3* genes indicated that genetic variation occurred during evolution. Notably, most of the *RPD3* genes were distributed on the opposite ends of the chromosomes in four cotton species (Fig. 3).

**Fig. 4:**
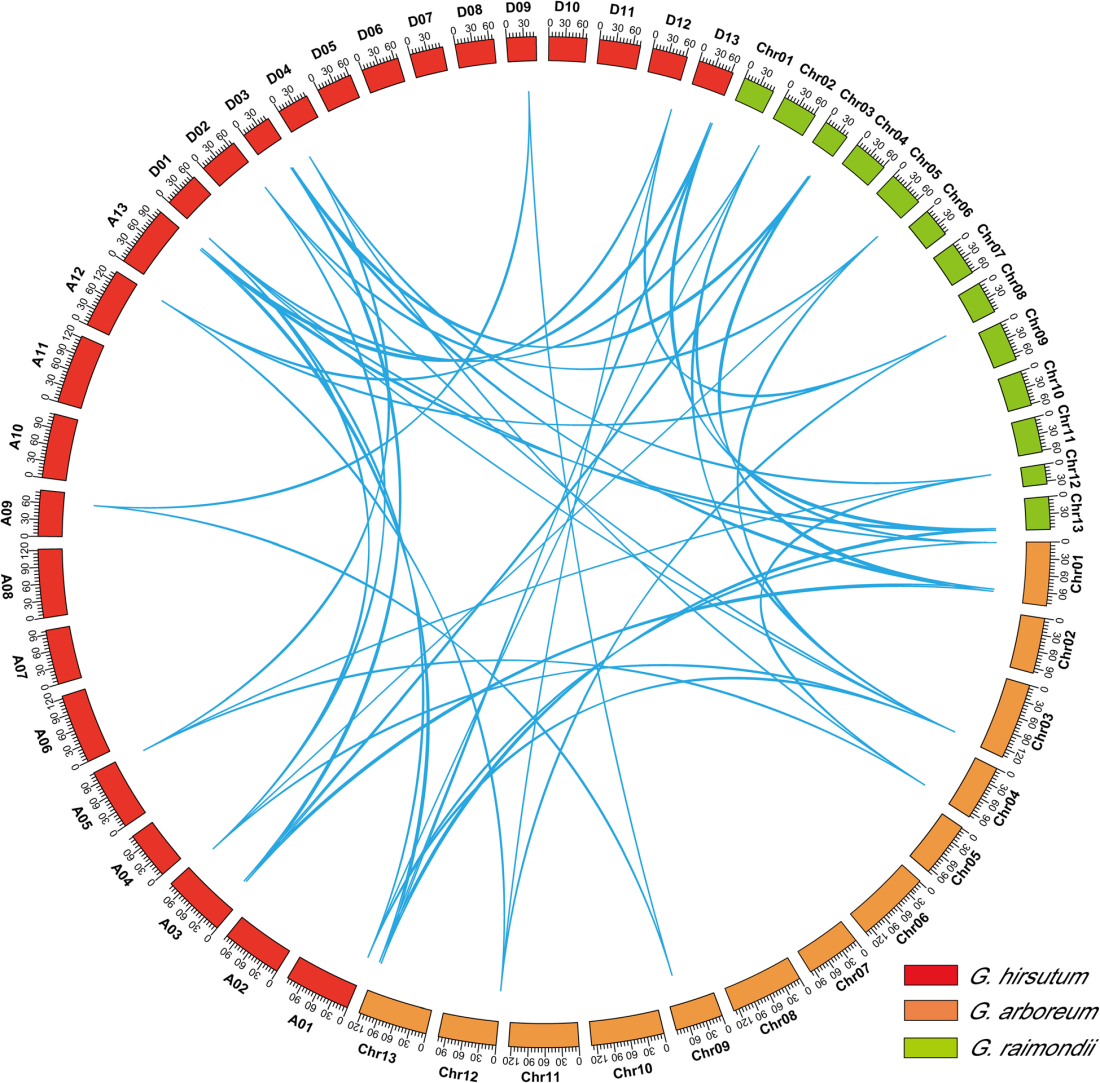
*RPD3* homologous gene pairs among *G. arboreum*, *G. raimondii* and *G. hirsutum*. Orange, blue and red represent chromosomes of *G. arboreum*, *G. raimondii* and *G. hirsutum*, respectively

In general, tandem and segmental duplication are two of the main reasons for gene family generation during evolution [21]. The analysis of gene duplication indicated that all *RPD3* family members were amplified only through segmental duplication (Additional file 6: Table S5), suggesting that segmental duplication played a vital role in the evolution of the *RPD3* gene family. The homologous gene pairs obtained by collinearity analysis among *RPD3* genes in *G. arboreum*, *G. raimondii*, and *G. hirsutum* were visualized using circular maps (Fig. 4). The Ka/Ks ratios of most homologous gene pairs were lower than one, indicating that purified selection was essential during the evolution of cotton *RPD3* genes, whereas the Ka/Ks ratios of two gene pairs (*GhHDA2*/*GaHDA3* and *GhHDA6/GaHDA6*) were more than 1, suggesting that these two pairs might have experienced positive selection pressure. The study also predicted the occurrence time of segmentally duplicated *RPD3* gene pairs by the formula “t = Ks/2r” (*r* = 2.6X10^− 9^) [22]. Except for the *GhHDA6*/*GaHDA6* gene pair, the other segmental duplication events of three cotton species might have occurred 0.6 to 144.44 million years ago (MYA) with an average time of 18.39 million years ago (Additional file 6: Table S5).

### Analysis of cis-elements in predicted promoter regions of *GhRPD3*

**Fig. 5:**
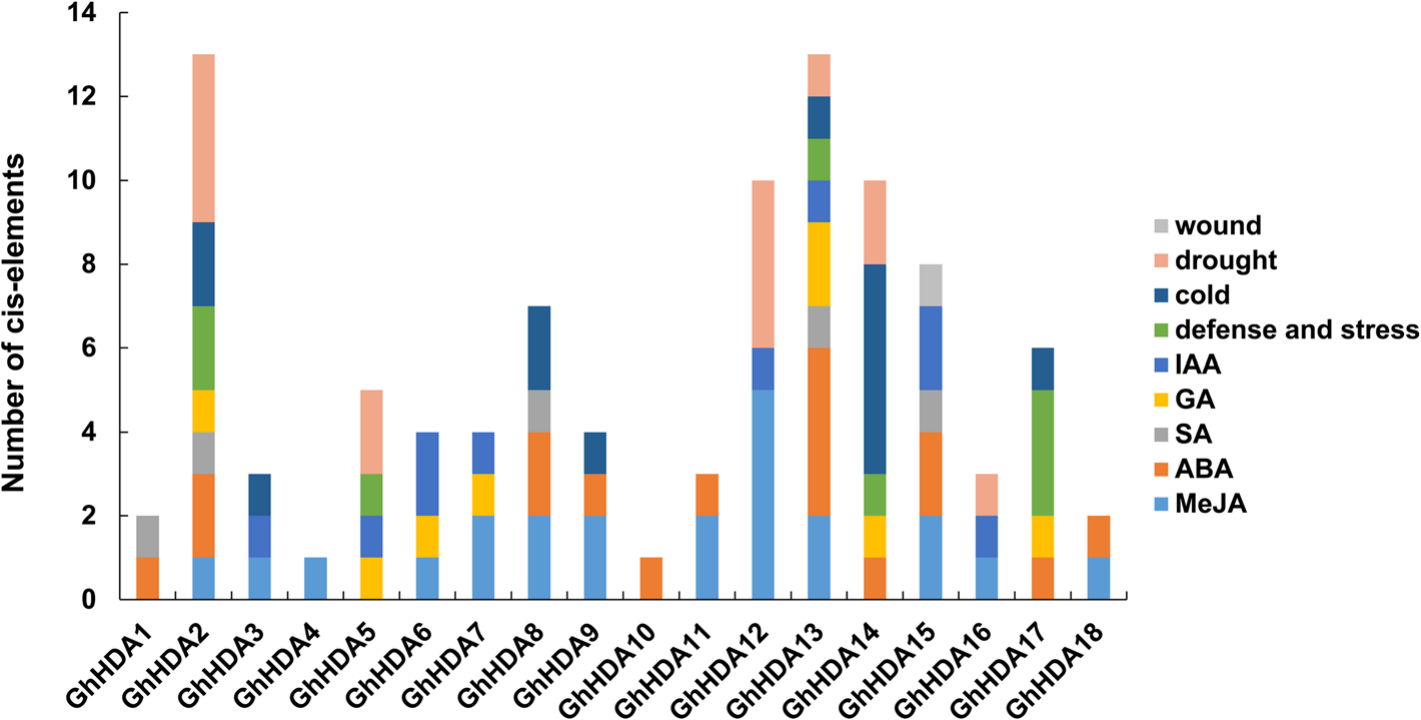
Cis-elements of *GhRPD3* genes in promoter regions. The numbers of different cis-elements are presented in the form of bar graphs, and similar cis-elements are exhibited with the same colors

To explore the possible regulatory functions of *GhRPD3* genes under various environmental stresses and hormone regulation pathways, the 2000-bp promoter regions of 18 *GhRPD3* genes were submitted to the PlantCARE database for the identification of putative stress-associated and plant hormone-related cis-elements. A total of 9 kinds of elements related to plant hormones, containing AuxRE-core (auxin), TGA-element (auxin), P-box (gibberellin), TATC-box (gibberellin), GARE-motif (gibberellin), CGTAC-motif (MeJA), TGACG-motif (MeJA), TCA-element (SA), and ABRE (ABA), and 4 kinds of elements responding to stresses, including TC-rich repeats (defense and stress responsiveness), MBS (drought), WUN-motif (wound) and LTR (cold stress), were predicted in the promoters of *GhRPD3* genes. As shown in Fig. 5, the promoters of some *GhRPD3* genes contained various hormone-responsive and stress-responsive components, such as *GhHDA2* (2 MBS, 2 LTR, 2 TC-rich repeats, 1 GARE-motif, 2 ABRE, 1 TGACG-motif) and *GhHDA13* (1 MBS, 1 LTR, 1 TC-rich repeats, 1 AuxRE-core, 2 GARE-motif, 1 TCA-element, 4 ABRE, 2 TGACG-motif). Among the 18 *GhRPD3* genes, there are large numbers of light-responsive elements distributed in their promoter regions (Additional file 7: Table S6). In addition, MeJA-responsive and ABA-responsive elements are more common than other hormone-related elements (Additional file 8: Figure S2). These results revealed that *GhRPD3* genes might be involved in MeJA and ABA hormone signaling pathways as well as response to environmental stresses.

### Expression profiles of *GhRPD3* genes in different tissues and under different abiotic stresses

**Fig. 6:**
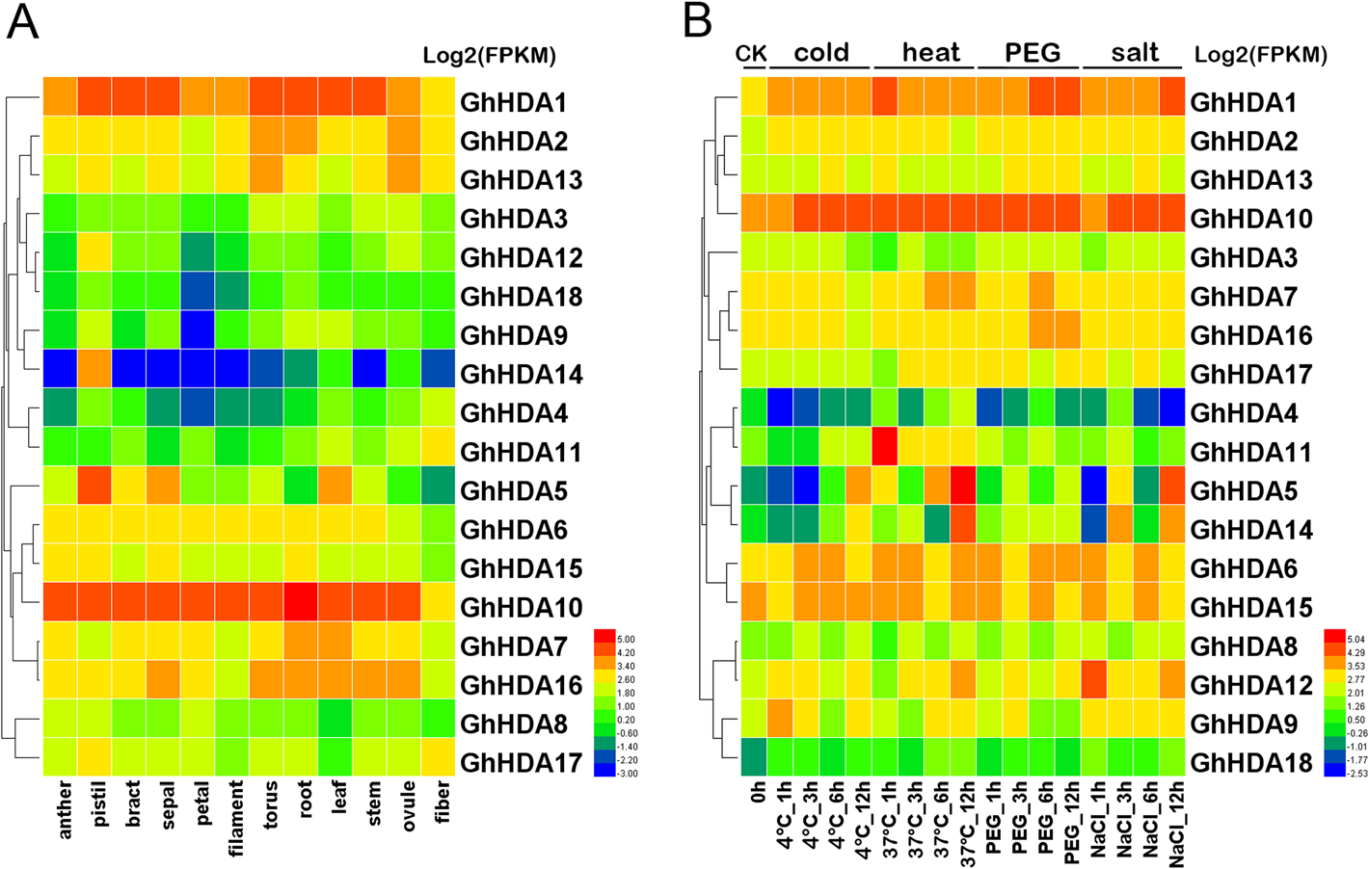
Expression patterns of *RPD3* genes in *G. hirsutum*. **a** and **b** represent the expression patterns of *GhRPD3* genes in different tissues (**a**) and under four different abiotic stresses (**b**), respectively. Gene names are shown on the right. Scale bars on the right represent the log2-transformed FPKM values of each gene

To understand the potential functions of *GhPRD3* genes in the growth and development of cotton, we studied their expression in various cotton tissues, including the anther, pistil, bract, sepal, petal, filament, torus, root, leaf, stem, ovules, and fibers, using publicly available transcriptomic data provided by Hu et al. [23]. Transcripts of all the *GhRPD3* genes were detected in at least three tissues with fragments per kilobase million (FPKM) ≥ 1. Furthermore, ten genes exhibited high expression levels in all selected tissues (Additional file 9: Table S7). These results indicated that *GhRPD3* genes are widely expressed in both reproductive organs and vegetative organs and thus might have multiple biological functions. After log2-conversion of FPKM values, the expression profiles of *GhRPD3* genes in different tissues are shown in Fig. 6a. Seven *GhRPD3* genes exhibited relatively high expression levels in at least eight tissues (log2-transformed FPKM value≥2.6); in particular, one pair of homologous genes (*GhHDA1*/*GhHDA10*) showed a high expression level in all the tissues with a similar expression pattern. Nevertheless, three *GhRPD3* genes (*GhHDA4*, *GhHDA14*, *GhHDA18*) were expressed at relatively low levels in at least eight tissues (log2-transformed FPKM value< 1), of which *GhHDA14* showed the lower expression in all tissues except for the pistil. These homologous gene pairs (*GhHDA1*/*GhHDA10*, *GhHDA4*/*GhHDA11*, *GhHDA2*/*GhHDA13*, *GhHDA6*/*GhHDA15*, *GhHDA7*/*GhHDA16*, and *GhHDA9*/*GhHDA18*) were located on At and Dt subgenomes and exhibited similar expression patterns. For example, homologous gene pairs (*GhHDA4*/*GhHDA11* and *GhHDA9*/*GhHDA18*) showed relatively low expression in all twelve tissues. The gene pair *GhHDA2*/*GhHDA13* exhibited relatively high expression in torus and ovule but relatively low expression in petals (Fig. 6a).

Based on the analysis of cis-elements in promoter regions and previous reports on *RPD3* genes in other plants, *GhRPD3* gens might respond to abiotic stresses. To test this hypothesis, we investigated the expression characteristics of 18 *GhRPD3* genes under cold, heat, PEG, and salt treatments using available transcriptomic data [23] (Fig. 6b). The expression of most *GhRPD3* genes were induced by the four stresses to varying degrees. *GhHDA1*, *GhHDA2*, *GhHDA6*, *GhHDA10*, *GhHDA12*, and *GhHDA18* showed upregulated expression under four stress treatments. However, one gene (*GhHDA4*) exhibited marked downregulation in the presence of the four abiotic stresses. Some genes can respond to one specific abiotic stress. For example, the expression of *GhHDA13* and *GhHDA16* was significantly induced by PEG treatment. Four genes (*GhHDA7*, *GhHDA11*, *GhHDA5*) showed upregulated expression under heat treatment. The expression of *GhHDA9* was significantly upregulated under cold and salt treatments. According to the results, we can conclude that *GhRPD3* genes play an essential role in response to abiotic stresses.

### Characterization of *GhRPD3* genes expression during flower bud differentiation

**Fig. 7:**
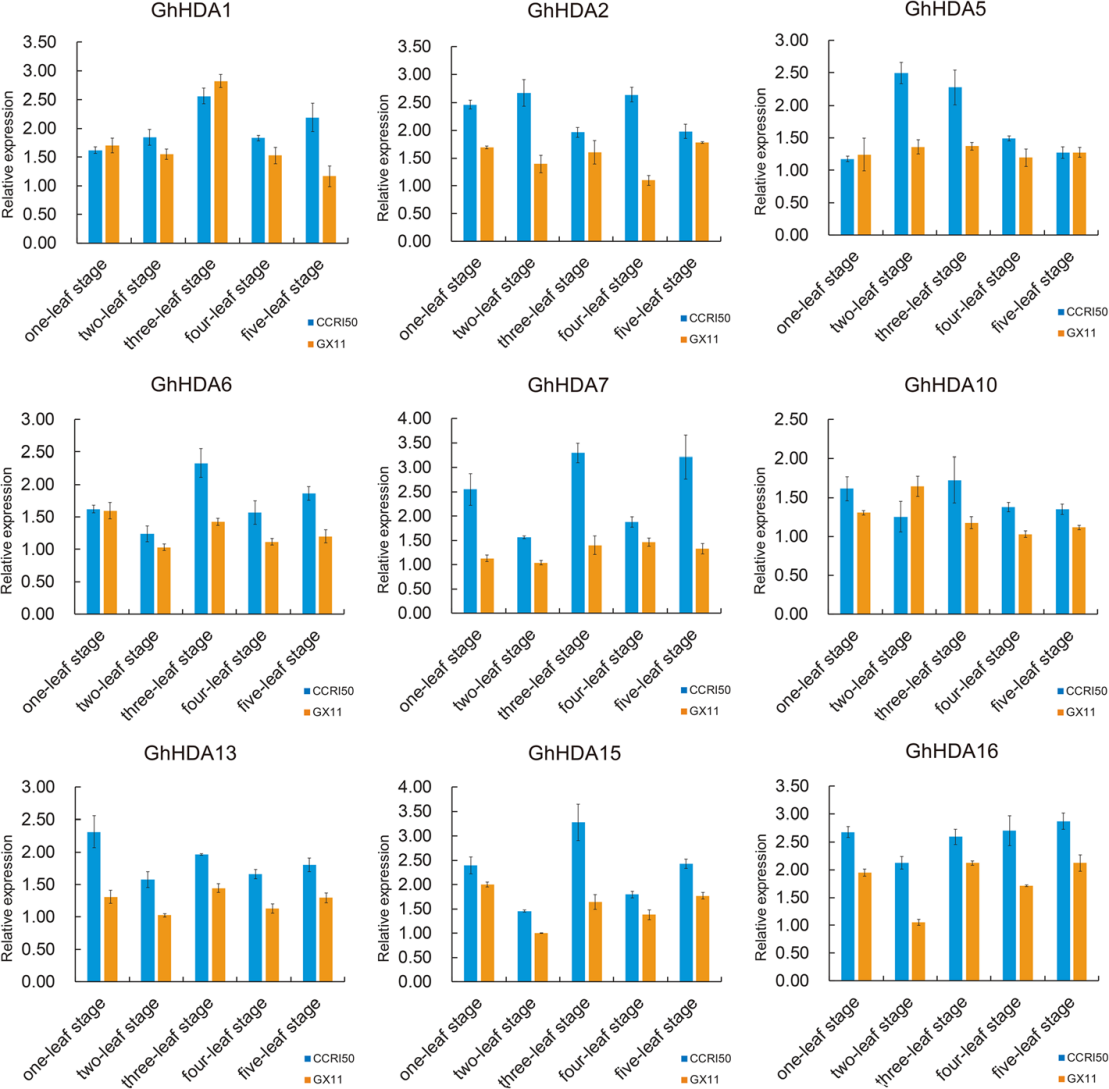
Expression levels of 9 *GhRPD3* genes between CCRI50 and GX11. Blue and orange bar graphs indicate the expression of early-maturity cotton (CCRI 50) and late-maturity cotton (GX11), respectively. The error bars show the standard deviation of three biological replicates

To explore expression differences of *GhRPD3* genes between early-maturity and late-maturity cottons during flower bud differentiation, we selected nine genes showing relatively high expression in floral organ tissues (anther, pistil, bract, sepal, petal, filament and torus) for qRT-PCR. The buds of an early-maturity variety (CCRI50) and a late-maturity variety (GX11) from the one-leaf to five-leaf stage were used for qRT-PCR (Fig. 7). The results revealed that more than half of these genes (5/9) possessed relatively higher expression in early-maturity cotton compared with late-maturity cotton during flower bud differentiation. *GhHDA5* showed marked differences at the two-leaf and three-leaf stages, and these two stages were regarded as the important period of flower bud differentiation. A homologous gene pair (*GhHDA6*/*GhHDA15*) located on At and Dt respectively, showed the same expression trend. Both of them presented the highest expression at three-leaf stage and then exhibited downregulated expression in next two stages in CCRI50. In addition, all nine genes showed relatively higher expression at the two-leaf or three-leaf stage in CCRI50 compared with GX11. The results showed that *GhRPD3* genes are associated with the early maturity of cotton.

### Responses of *GhRPD3* genes to MeJA and ABA treatment

**Fig. 8:**
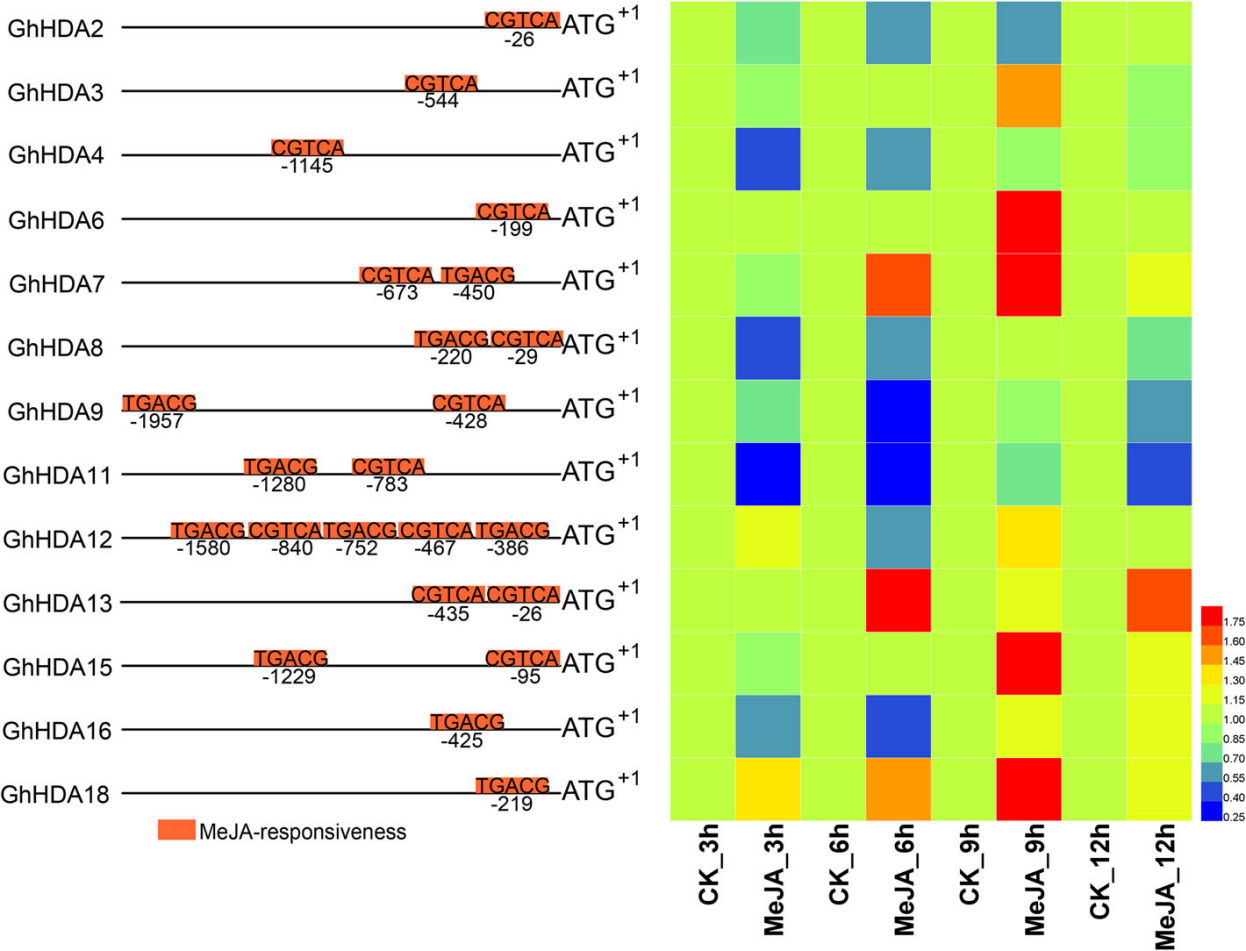
Expression profiles of 13 *GhRPD3* genes under MeJA treatment. Orange boxes represent the MeJA-responsive elements of 13 *GhRPD3* genes in the promoter regions (left). The expression changes of 13 *GhRPD3* genes under MeJA treatment are shown using a heatmap (right). qRT-PCR was carried out with three technical and three biological replicates. Relative expression levels of each gene were calculated after normalizing the expression level in CK (water) to 1.0

**Fig. 9:**
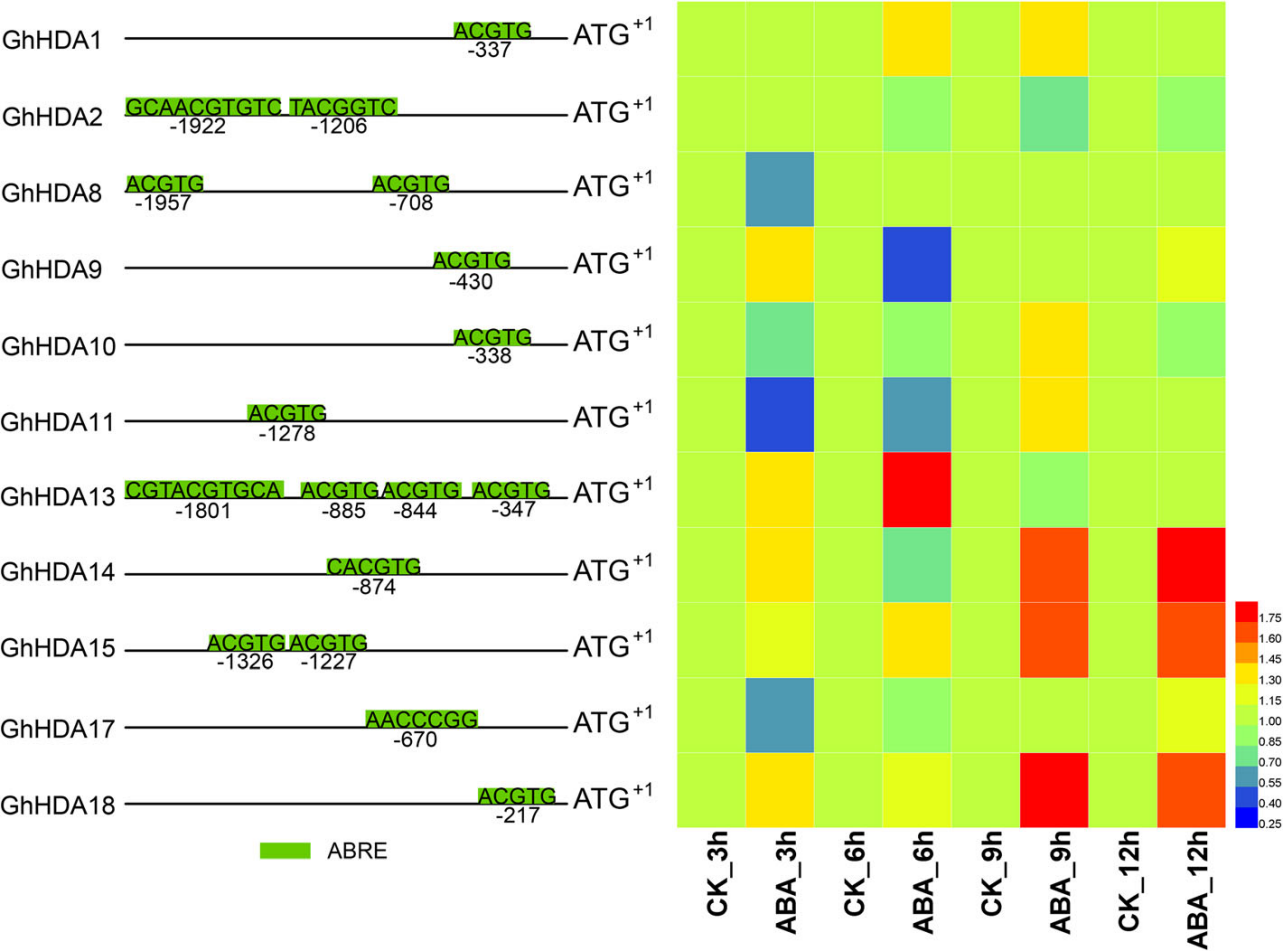
Expression patterns of 11 *GhRPD3* genes under ABA treatment. Green boxes represent the ABRE of 11 *GhRPD3* genes in the promoter regions (left). The expression changes of 11 *GhRPD3* genes under ABA treatment are shown using a heatmap (right). qRT-PCR was conducted with three technical and three biological replicates. Relative expression levels of each gene were calculated after normalizing the expression level in CK (water) to 1.0

MeJA and ABA play important roles in plant stress resistance. To further explore the possible functions of *GhRPD3* genes, we selected the *GhRPD3* genes containing MeJA- and ABA-responsive elements in the predicted promotors to analyze their expression characteristics under MeJA and ABA treatment by qRT-PCR (Figs. 8 and 9). Most *GhRPD3* genes (8/13) were markedly upregulated at 9 h after MeJA treatment. Three genes (*GhHDA7*, *GhHDA13*, and *GhHDA18*) exhibited significantly upregulated expression at three or more time points, whereas four genes (*GhHDA2*, *GhHDA8*, *GhHDA9*, and *GhHDA11*) showed marked transcriptional downregulation at least three time points after MeJA treatment (Fig. 8). More than half of the *GhRPD3* genes (6/11) were significantly upregulated at 9 h after ABA treatment. Three *GhRPD3* genes (*GhHDA14*, *GhHDA15*, and *GhHDA18*) showed relatively high expression at three or more time points, whereas three *GhRPD3* genes (*GhHDA10*, *GhHDA11*, and *GhHDA17*) showed early downregulated and then upregulated expression patterns under ABA treatment (Fig. 9). The results showed that the exogenous application of MeJA and ABA significantly induced the transcription of most *GhRPD3* genes containing MeJA-responsive and ABRE elements in their promoter regions.

## Discussion

Among the several histone modifications, histone acetylation plays an essential role in plant growth and development [24]. Histone acetylation and deacetylation are catalyzed by histone acetyltransferases (HATs) and histone deacetylases (HDACs), respectively [20]. In plants, HDACs are involved in a variety of biological processes associated with plant growth and development [25]. Within the superfamily of HDACs, the *RPD3* gene family is the most studied and is crucial in plant development and physiological processes, including flowering time, abiotic stress response, female gametophyte and embryo development, senescence, seed germination, and plant hormone signal response [12–14, 19, 26–28]. To date, although a few studies have analyzed the function of the *RPD3* family members in *G. hirsutum*, they focus primarily on the roles of *GhHDA5* in fiber initiation, and there are no systematic reports [29]. To explore the characteristics of *RPD3* family members and understand the roles played by cotton *RPD3* genes in cotton growth and development, we conducted an integrated analysis of the *RPD3* gene family in cotton, containing their phylogenetic relationships, exon-intron structures, conserved motifs, chromosomal distributions, duplication events, expression patterns in different tissues and in the presence of abiotic stresses or MeJA and ABA treatment.

### Phylogeny, gene structure, and expansion of *RPD3* genes in cotton

A total of 18, 18, 9 and 9 *RPD3* genes were identified by genome-wide identification in *G. hirsutum*, *G. barbadense*, *G. aboreum*, and *G. raimondii*, respectively. The number of *RPD3* cotton genes in diploid cotton was half of that in tetraploid cotton, suggesting that the deletion of *RPD3* genes did not happen in allotetraploids, which is not in agreement with the higher ratio of gene deletion in allotetraploids [30]. According to the *AtRPD3* genes, 108 *RPD3* genes from nine species were classified into four groups (Fig. 1), similar to the previous classification of *Arabidopsis* [7].

The conservation of biological functions might be related to the conservation of gene structure [31]. To investigate the conservation of the *RPD3* gene sequences, exon-intron structure and conserved motifs were analyzed. Exon-intron structure analysis showed that the exon numbers of *RPD3* genes in cotton was highly diverse, ranging from 3 to 17 (Additional file 4: Table S3), which might be associated with the diversification of their functions. Notably, the gene structure and motif arrangement were different among the four subfamilies, whereas they were highly conserved on the same branch, indicating that the *RPD3* genes (especially the members on the same branch) might preform conserved functions in the growth of cotton. The cotton *RPD3* genes exhibited similarities and differences in exon-intron structures and motifs, which might be associated with conservation and subfunctionalization caused by gene duplication during the evolution of the cotton *RPD3* gene family.

According to the evolutionary history of cotton, tetraploid cotton was formed by the hybridization of two diploid cottons with subsequent polyploidization [31]. To investigate the evolutionary relationships of predicted *RPD3* genes between two diploid genomes and subgenomes in allotetraploids, we analyzed their chromosomal distributions and gene duplication events. The results showed that the chromosomal distribution of *RPD3* genes in *G. arboreum* and the corresponding A subgenome of *G. hirsutum* was not identical, whereas the chromosomal location of *RPD3* genes in *G. raimondii* and the corresponding D subgenome of *G. hirsutum* was highly consistent (Fig. 3), illustrating strong conservation during *RPD3* gene family evolution. The analysis of gene duplication showed that segmental duplication was essential for the expansion of *RPD3* family members (Additional file 6: Table S5). According to previous genomic studies of cotton, A and D genome diploid cottons began to differentiate from a common ancestor 5–10 MYA [23]. Subsequently, *G. hirsutum* evolved from the hybridization of two diploid cottons approximately 1–2 MYA [22]. In *G. hirsutum*, the deduced divergence times of most *RPD3* homologous gene pairs varied from 5.66 to 11.06 MYA (Additional file 6: Table S5), which was accompanied by the divergence of A and D ancestral genomes. Ka/Ks ratio analysis indicated that the segmentally duplicated gene pairs might perform similar functions based on purified selection in functional segregation [32].

### Functional analysis of *GhRPD3* genes in upland cotton

Flowering time, an important indicator of precocity, is also influenced by the timing of flower bud differentiation, which is a physiological and morphological marker of the transformation from vegetative growth to reproductive growth [33, 34]. Previous studies showed that the flower bud differentiation period of early-maturity cotton varieties occurs earlier than that of late-maturity cotton varieties, and early-maturity cotton generally begins flower bud differentiation when three true leaves are completely flattened [33]. In *Arabidopsis*, at least 4 *RPD3* genes have been associated with flowering time; *AtHDA6* has been related with the autonomous pathway of four flowering-promoting pathways and regulates flowering time by interacting with *FLD* (*Flowering LOCUS D*) [13, 26]; *HDA5* regulates flowering time by repressing the expression of *FLC* (*FLOWERING LOCUS C*) and *MAF1*. In addition, *HDA5* and *HDA6* might form an HDAC complex with *FLD* and FVE to control flowering time in *Arabidopsis* [27]. In short days, *AtHDA9* represses the flowering-promoting gene *AGL19* (*AGAMOUS-LIKE 19*) regulated by photoperiod [35] and the downregulation of *AtHDA19* causes delayed flowering, flower abnormalities, embryonic defects, and seed set reduction [36]. In recent studies, *GhHDA5*, similar to *AtHDA5* in *Arabidopsis*, exhibited higher expression at − 1 and 0 DPA, and RNAi-repressed *GhHDA5* lines showed delayed flowering, suggesting that it may be involved in cotton fiber initiation and flowering time [29]. As the homologous genes of these four *AtRPD3* genes related to flowering time in cotton, the gene pairs *GhHDA2*/*GhHDA13*, homologous to *AtHDA6*, and *GhHDA6*/*GhHDA15*, homologous to *AtHDA9*, showed markedly higher expression in early-maturity cotton compare with late-maturity cotton during all five stages of flower bud differentiation. The other five *GhRPD3* genes we selected exhibited relatively higher expression at the two-leaf or three-leaf stage of early-maturity cotton (Fig. 7), indicating that *GhRPD3* genes are helpful for improving the molecular breeding of early-maturity cotton.

Cis-elements in promoter regions play important roles in gene expression regulation. In general, gene expression depends on the presence or absence of these elements [37]. In previous studies, multiple lines of evidence have revealed that *RPD3* members play essential roles in response to various stresses or plant hormones in *Arabidopsis*, rice, maize, *Populus trichocarpa*, and others [13, 17, 38]. To better understand the regulation of *GhRPD3* genes under different environmental conditions, we investigated the cis-elements in their promoter regions. Nine kinds of plant hormone-related elements and 4 kinds of stress-responsive regulatory elements were identified in the presumed promoters of *GhRPD3* genes (Fig. 5). Such a wide range of cis-acting elements is consistent with the published studies on the multifunctional roles *RPD3* genes play in plant growth [7, 8]. Based on the expression patterns of *GhRPD3* genes under four abiotic stresses, almost all of the 18 genes were significantly induced by all four stresses or by a specific treatment (Fig. 6b), fully illustrating that *GhRPD3* genes can respond to abiotic stresses.

MeJA and ABA not only regulate plant growth and development but also participate in plant defense response to environmental stress such as mechanical injury, disease, and osmotic stress [39, 40]. Previous studies showed that the expression of *HDA705*, *HDA702*, and *HDA704* could be induced in rice by JA or ABA [19, 20]. The transcription of *AtHDA6*, *AtHDA19*, and *AtHDA9* has been reported to be increased after ABA or JA treatment in *Arabidopsis*. In *AtHDA6* mutants, *axe1–5* and *HDA6-RNAi* plants, the expression of JA-responsive and ABA-responsive genes was significantly downregulated, and the mutants exhibited hypersensitivity to ABA during seed germination [13, 41]. *AtHDA19–1*, a T-DNA insertion mutant, showed similar phenotypes as *AtHDA6* mutant in response to ABA and in gene expression, whereas overexpression of *AtHDA19* enhanced the expression of defense genes synergistically induced by JA and ethylene [17, 38]. According to the analysis of cis-elements in predicted *GhRPD3* promotors, we selected the *GhRPD3* genes containing MeJA-responsive and ABA-responsive elements in the predicted promoters to analyze their expression characteristics under MeJA and ABA treatment by qRT-PCR. The results revealed that exogenous MeJA and ABA application significantly induced the transcription of most *GhRPD3* genes at different time points (Figs. 8 and 9). *GhHDA13*, similar to *AtHDA6*, was markedly upregulated after treatment with MeJA and ABA. The significant induction of *GhHDA13* expression under cold and PEG treatment might be related to the presence of cold and drought-responsive cis-elements in its predicted promoter. As the homologous gene of *AtHDA9*, *GhHDA15* not only showed relatively high transcriptional levels after ABA treatment at all four stages but also exhibited significantly upregulated expression under MeJA treatment at 9 h and 12 h. Some *RPD3* genes were less studied in other plants, but they could respond to MeJA and ABA treatment in upland cotton. For example, *GhHDA18* positively responded to ABA and MeJA treatment at all four stages, and its expression was slightly induced under stress conditions. The homologous gene pair *GhHDA7*/*GhHDA16* showed higher expression at 9 h and 12 h after exogenous application of MeJA, and their expression could be induced by PEG treatment. The homologous gene pair *GhHDA8*/*GhHDA17* showed lower expression in the early stages of ABA treatment. The expression patterns of *RPD3* genes indicated that they might participate in abiotic stress responses via ABA and MeJA signaling pathways.

On the basis of our expression profile analysis of *RPD3* genes in upland cotton, *GhHDA1*, *GhHDA2*, *GhHDA6*, *GhHDA10*, and *GhHDA13* showed relatively high expression levels in most of the investigated tissues and in early-maturity cotton during flower bud differentiation. These genes also played essential roles in response to MeJA, ABA, and abiotic stresses, which is consistent with the previous extensive evidences showing that plant histone deacetylases play vital roles in plant developmental processes and responses to various environmental stresses [7, 17, 26, 27, 41, 42]. Additionally, other *GhRPD3* genes also performed specific functions in cotton development, laying the foundation for further functional verification of *RPD3* genes in upland cotton.

## Conclusions

In this study, a total of 108 *RPD3* genes were detected in nine species by genome-wide identification. These genes were divided into four subgroups according to the classification in *Arabidopsis*. The exon-intron structure and conserved motif analysis of 54 cotton *RPD3* genes showed that significant differences exist among the four subfamilies, whereas they are highly conserved on the same branch, indicating that cotton *RPD3* genes on the same branch might perform similar functions in cotton growth and development. The chromosomal distributions of cotton *RPD3* genes revealed conserved gene numbers and chromosomal locations between diploid and tetraploid cotton species. Gene expression analysis showed that most *GhRPD3* genes had relatively high expression in floral organs and exhibited the higher expression in CCRI50 compared with GX11 during flower bud differentiation. In addition, the expression of *GhRPD3* genes could be significantly induced by one or more abiotic stresses or exogenous application of MeJA and ABA. These results revealed that *GhRPD3* genes might be involved in flower bud differentiation and resistance to abiotic stresses in cotton, which provides a basis for further functional verification of *GhRPD3* genes in cotton development and a foundation for breeding better early-maturity cotton cultivars in the future.

## Methods

### Plant materials and treatments

Two *G. hirsutum* cultivars (CCRI50, GX11) were grown under standard field environments (5 rows, each 8 m long and 0.8 m wide) in Anyang, Henan province, China. CCRI 50 is an early-maturity cotton variety with initial flowering time of 60 days, and GX11 is a late-maturity cotton cultivar with initial flowering time of 70 days. The buds of two cotton cultivars were collected from the one-leaf to five-leaf stage to analyze the expression differences of *GhRPD3* genes between CCRI 50 and GX11 cultivars during flower bud differentiation.

TM-1 was planted in a climate-controlled greenhouse with a suitable growing environment (light/dark cycle: 16 h at 28 °C/8 h at 22 °C). Four-week seedlings showing two flat true leaves were sprayed with 100 mM MeJA, 200 mM ABA, and water as a blank control to explore the responses to MeJA and ABA treatment. After exogenous application of plant hormones and water, we isolated the leaves of three seedlings from every treatment at 3 h, 6 h, 9 h, and 12 h and promptly froze these samples in liquid nitrogen for RNA extraction.

### Identification and sequence retrieval of *RPD3* family members

The HMM file (PF00850) of the conserved Hist_deacetyl domain was downloaded from the Pfam database (https://pfam.xfam.org/) [43]. Putative *RPD3* proteins of *G. arboreum* (CRI_1.0) [44], *G. raimondii* (JGI_v2.1) [45], *G. hirsutum* (HAU_v1) [46] and *G. barbadense* (HAU_v1) [46] from the CottonFGD (http://www.cottonfgd.org/) [47] were searched using the hidden Markov model profile of the HMMER 3.0 software with an E value threshold of 1e-10 [48]. The study also searched against the *Arabidopsis* genome (Araport_11) [49] obtained from the TAIR website (https://www.arabidopsis.org/) and the published genomes of *Oryza sativa L.* (JGI_v7.0), *Theobroma cacao* (JGI_V2.1), *Populus trichocarpa* (JGI_v3.1), and *Zea mays L.* (JGI_v4) downloaded from phytozome_V13 (https://phytozome.jgi.doe.gov/pz/portal.html) [50] using the Hist_deacetyl HMM file. After obtaining the ID number of the possible genes in these nine protein databases, the *RPD3* protein sequences of different species were extracted from the formatted protein databases using blast (ncbi-blast-2.6.0 + −× 64-win64.tar). The normal model of the SMART database (http://smart.embl-heidelberg.de/) was employed to verify each predicted *RPD3* protein with a Hist_deacetyl domain, and proteins that did not contain the conserved domain were removed [51]. These *RPD3* genes were named following a rule that short for species names.

The identified *RPD3* protein sequences were submitted to the online program Pepstats (https://www.ebi.ac.uk/Tools/seqstats/emboss_pepstats/) [52] and CELLO v2.5 (http://cello.life.nctu.edu.tw/) [53] to predict their amino acid length, theoretical molecular weight (Mw), isoelectric point (pI), and subcellular localization.

### Multiple alignment and phylogenetic analysis of *RPD3* proteins

Multiple alignment of all the presumed *RPD3* protein sequences from the nine plant species including *G. raimondii*, *G. arboreum*, *G. hirsutum*, *G. barbadense*, *A. thaliana*, *T. cacao*, *P. trichocarpa*, *Oryza sativa*, and *Zea mays* was performed using the ClustalW program with default parameters [54]. The alignment result was employed to construct an unrooted phylogenetic tree using the neighbor-joining (NJ) method of MEGA 7.0 program and 1000 bootstrap repetitions were used to increase the reliability of interior branches [55].

### Chromosomal distribution, gene structure, and conserved motif analysis

The physical positions of *GhRPD3*, *GbRPD3*, *GaRPD3*, and *GrRPD3* genes on chromosomes were confirmed based on the genome annotation GFF3 files obtained from the CottonFGD website, and the distribution of cotton *RPD3s* were visualized using TBtools [47, 56]. The GFF3 files of *RPD3* genes in four cotton species were submitted to the online toolkit GSDS 2.0 (http://gsds.cbi.pku.edu.cn/) for analysis and visualization of the exon-intron structure [57]. The online program MEME 5.0.5 (http://meme-suite.org/tools/meme) was employed to detect the conserved motifs of cotton *RPD3* proteins with the following optimized parameters: maximum number of motifs, 10; the optimum width of each motif, 6–50 aa; and E value, 1e-5 [58].

### Gene duplication events and selection pressure

This study used BLASTp search (E-value <1e-10) to align protein sequences in three cotton species, and the MCScanX program in TBtools was employed to perform genome collinearity analysis based on the BLASTp results [56, 59].. The circular maps of identified *RPD3* gene pairs in three cotton species were displayed using the circos program [60]. The adjacent *RPD3* family members on a single chromosome were considered to be tandem duplicated genes [61]. The coding sequences of *RPD3* homologous gene pairs were used to calculate the ratios of nonsynonymous (Ka) substitutions and synonymous (Ks) substitutions by the NG methods of TBtools to evaluate the selection pressure of these gene pairs [56, 62]. Normally, Ka/Ks < 1 indicates purifying selection; Ka/Ks = 1 indicates neutral selection; and Ka/Ks > 1 indicates positive selection. The divergence times of the homologous gene pairs were estimated using the formula t = ks/2r, with *r* = 2.6X10–9 representing neutral substitution [63].

### Analysis of cis-elements in *GhRPD3* promoter regions

The *GhRPD3* promoter regions containing 2000 bp of DNA upstream of the initiation codon (ATG) were extracted from the *G. hirsutum* genome database downloaded from CottonFGD (https://cottonfgd.org/). The regulatory elements in the *GhRPD3* promoter regions were predicted using the online tool PlantCARE (http://bioinformatics.psb.ugent.be/webtools/plantcare/html/) [64].

### Gene expression pattern analysis

Primary RNA-seq data of *G. hirsutum* TM-1 were obtained from the NCBI Sequence Read Archive (SRA: PRJNA490626) (https://www.ncbi.nlm.nih.gov/bioproject/PRJNA490626) [23]. Transcriptomic reads were mapped to the *G. hirsutum* genome using TopHat2 with the default parameters [65], and gene expression was calculated in fragments per kilobase million (FPKM) by the cufflinks program [66]. The FPKM values of TM-1 in twelve different tissues (anther, pistil, bract, sepal, petal, filament, torus, root, leaf, stem, ovule, and fiber) and under four treatments (heat, cold, PEG, and salt) were obtained. The relative data of *GhRPD3* genes were normalized by log2 transformation to investigate their expression patterns. The expression characteristics of *GhRPD3* genes among all twelve tissues and under four abiotic stresses were displayed with HemI 1.0.3.7 software [67].

### RNA extraction and quantitative RT-PCR (qRT-PCR) experiments

Cotton buds collected at different stages and leaves taken after MeJA and ABA treatment were frozen in liquid nitrogen, and then a mortar and pestle were used to grind the samples into fine powder [68]. Depending on the operating instructions, total RNA of these samples was extracted using the Tiangen RNA-prep Pure Plant kit (Tiangen, China), and then 1 μg of total RNA was used as template to reverse-transcribe first-strand cDNA using the PrimeScript RT Reagent kit (Takara, Japan); cDNA was diluted five-flod for further experiments. The cotton histone-3 gene (AF024716) was used as an internal control [69], and specific primers for qRT-PCR analysis of *GhRPD3* genes were designed using Oligo 6.0 software and are shown in Additional file 10: Table S8. A total volume of 20 μL containing 10 μL 2 × UltraSYBR Mixture, 0.4 μL of each primer (10 μM), 2 μL cDNA, and 7.2 μL ddH2O was employed to conduct qRT-PCR on an ABI 7500 real-time PCR system (Applied Biosystems, USA) using UltraSYBR Mixture (Low ROX) (Cwbio, China) with three technical repetitions and three biological replicates. The following detailed run method was used: step 1, primal denaturation of 10 min at 95 °C; step 2, 40 cycles of 10 s at 95 °C, 30 s at 60 °C, and 32 s at 72 °C; step 3, melting curve analysis. The relative expression of *RPD3* genes was calculated using the 2^-△△CT^ method [70].

## Supplementary information

**Additional file 1: Table S1.** Detailed parameters of the *RPD3* proteins in nine species.

**Additional file 2: Table S2.** Location of Hist_deacetyl domain in cotton *RPD3* proteins.

**Additional file 3: Figure S1.** The conserved Hist_deacetyl domain of cotton *RPD3* proteins. (a) Phylogenetic relationships of cotton *RPD3* proteins and subfamilies of these proteins are exhibited using MEGA 7.0 with the neighbor-joining (NJ) method; (b) Conserved domains of 54 cotton *RPD3* proteins. The green boxes represent the Hist_deacetyl domain.

**Additional file 4: Table S3.** Numbers of introns and exons of cotton *RPD3* genes.

**Additional file 5: Table S4.** Chromosomal location of *RPD3* genes in *G. arboreum*, *G. raimondii*, *G. barbadense* and *G. hirsutum.*

**Additional file 6: Table S5.** Ka/Ks ratios and occurrence times of segmentally duplicated *RPD3* gene pairs of three cotton species. When Ks was equal to 0, Ka/Ks ratios was marked as Ka> > Ks.

**Additional file 7: Table S6.** Cis-acting elements in the promoters of *GhRPD3* genes.

**Additional file 8: Figure S2.** The ratios of 5 kinds of plant hormone-related cis-elements. Five different kinds of plant hormone-related cis-elements are represented by different colors.

**Additional file 9: Table S7.** The FPKM value of *GhRPD3* genes in different tissues and under four different abiotic stresses.

**Additional file 10: Table S8.** Specific primers of *GhRPD3* genes for qRT-PCR.

## Data Availability

The data included in this article and the additional files are available. The transcriptome datasets of *G. hirsutum* TM-1 are under the accession number in PRJNA490626 NCBI.

## Abbreviations

HDACs: Histone deacetylases
HATs: Histone acetyltransferases
RPD3/HDA1: Reduced potassium dependency 3/histone deacetylase 1
ABA: Abscisic acid
JA: Jasmonic acid
SA: Salicylic acid
MeJA: Methyl jasmonate
ABRE: Abscisic acid responsiveness elements
MW: Molecular weight
GFF: General feature format
FPKM: Fragments per kilobase million
MYA: Million years ago
qRT-PCR: Quantitative real time polymerase chain reaction
