# Supplementary figures and images for: Genome-wide identification and expression patterns analysis of the RPD3/HDA1 gene family in cotton
Source: BMC Genomics. 2020 Sep 18;21:643. doi: 10.1186/s12864-020-07069-w (PMC7501681; doi:10.1186/s12864-020-07069-w)

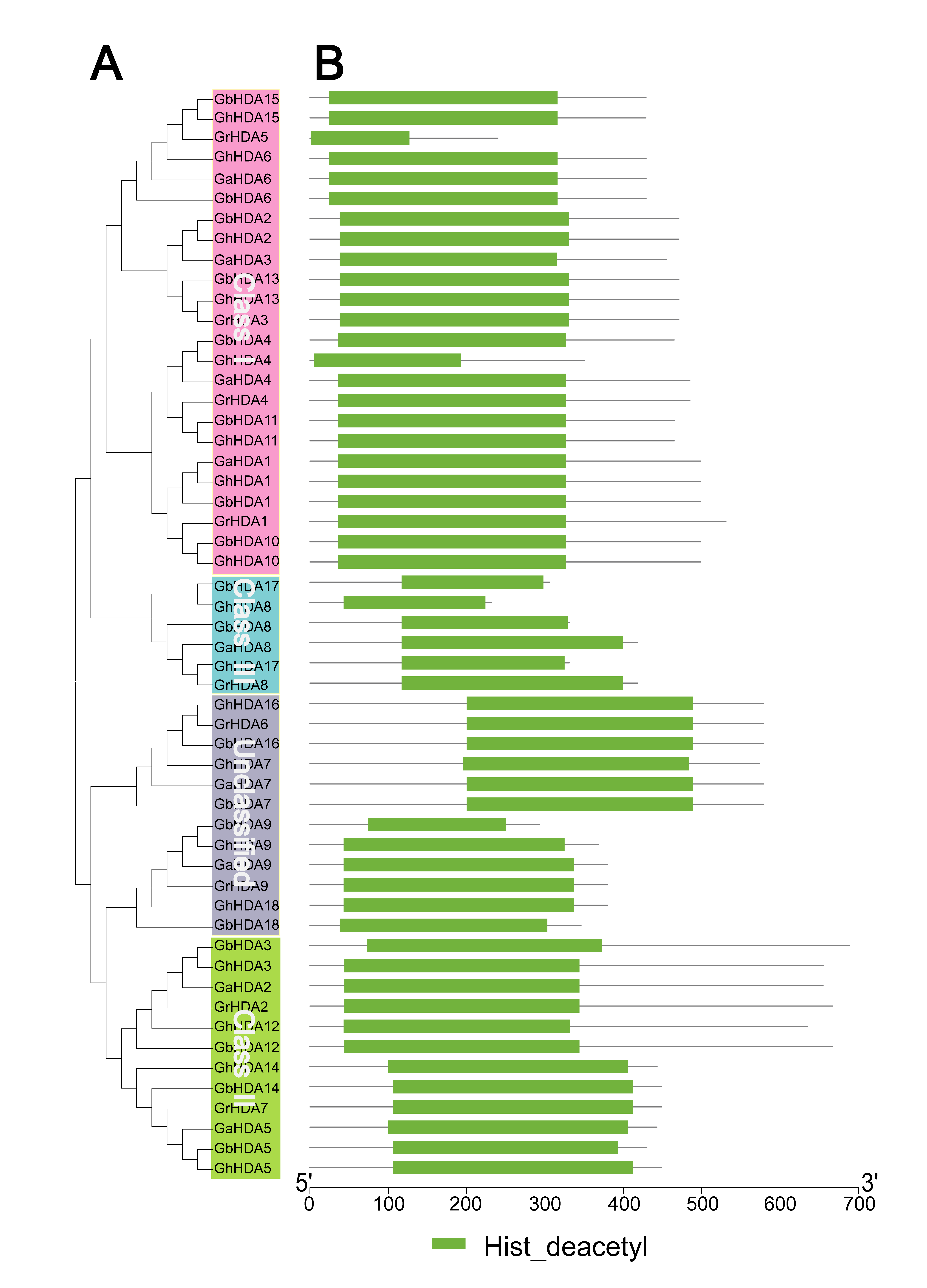

Supplement: Supplementary file 3 — Additional file 3: Figure S1. The conserved Hist_deacetyl domain of cotton RPD3 proteins. (a) Phylogenetic relationships of cotton RPD3 proteins and subfamilies of these proteins are exhibited using MEGA 7.0 with the neighbor-joining (NJ) method; (b) Conserved domains of 54 cotton RPD3 proteins. The green boxes represent the Hist_deacetyl domain. [file 12864_2020_7069_MOESM3_ESM.tif]

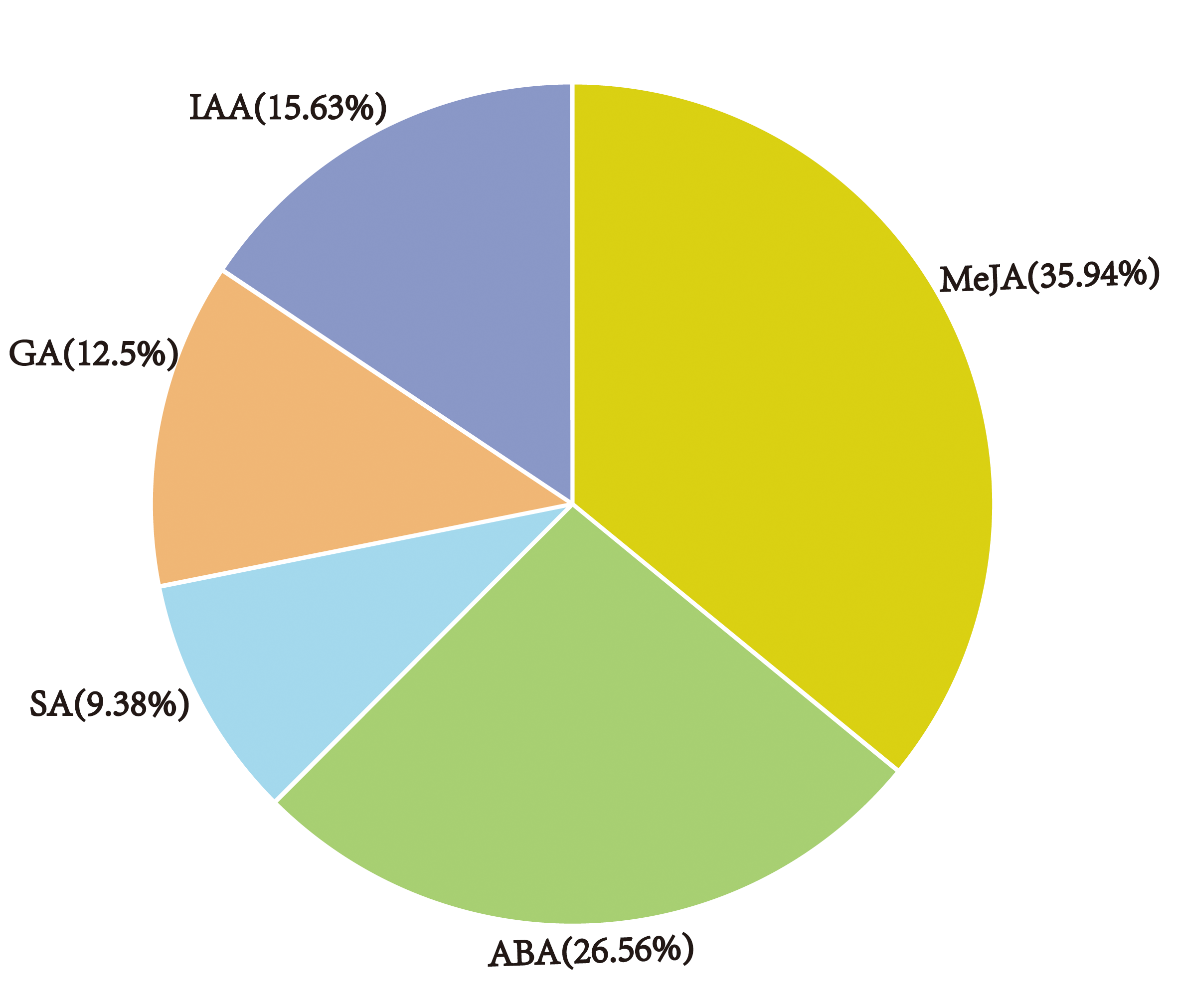

Supplement: Supplementary file 8 — Additional file 8: Figure S2. The ratios of 5 kinds of plant hormone-related cis-elements. Five different kinds of plant hormone-related cis-elements are represented by different colors. [file 12864_2020_7069_MOESM8_ESM.tif]
